# A spatio-temporal geodatabase of mortalities due to respiratory tract diseases in Tehran, Iran between 2008 and 2018: a data note

**DOI:** 10.1186/s13104-020-05319-4

**Published:** 2020-10-07

**Authors:** Elahe Pishgar, Alireza Mohammadi, Nasser Bagheri, Behzad Kiani

**Affiliations:** 1 Department of Human Geography and Logistics, Faculty of Earth Science, Shahid Beheshti University, Tehran, Iran; 2 Department of Geography & Planning, Faculty of Social Sciences, University of Mohaghegh Ardabili, Ardabil, Iran; 3 Visualization and Decision Analytics (VIDEA) Lab, Centre for Mental Health Research, Research School of Population Health, College of Health and Medicine, The Australian National University, Canberra, Australia; 4 Department of Medical Informatics, School of Medicine, Mashhad University of Medical Sciences, Mashhad, Iran

**Keywords:** Respiratory tract diseases, Mortality, Space–time analysis, Clustering, Geographical information systems, Tehran, Iran

## Abstract

**Objectives:**

Respiratory tract diseases (RTDs) are among the top five leading causes of death worldwide. Mortality rates due to respiratory tract diseases (MRRTDs) follow a spatial pattern and this may suggest a potential link between environmental risk factors and MRRTDs. Spatial analysis of RTDs mortality data in an urban setting can provide new knowledge on spatial variation of potential risk factors for RTDs. This will enable health professionals and urban planners to design tailored interventions. We aim to release the datasets of MRRTDs in the city of Tehran, Iran, between 2008 and 2018.

**Data description:**

The Research data include four datasets; (a) mortality dataset which includes records of deaths and their attributes (age, gender, date of death and district name where death occurred), (b) population data for 22 districts (age groups with 5 years interval and gender by each district). Furthermore, two spatial datasets about the city are introduced; (c) the digital boundaries of districts and (d) urban suburbs of Tehran.

## Objective

Respiratory tract diseases (RTDs) have a major impact on disease burden including death across the world [1]. RTDs are among the top five leading causes of death worldwide and Covid-19 has brought RTDs in focus [2, 3]. More than 9.5 million deaths globally are attributed to RTDs with four million premature death per year [3, 4]. Previous Research demonstrated possible links between mortality rates due to respiratory tract diseases (MRRTDs) and environmental risk factors [5].

Urbanization, air pollution and sedentary lifestyle are associated with an increased risk of asthma and respiratory diseases in urban areas compared to the rural regions [6]. Tehran is the capital city of Iran and is the 22nd most populous city in the world with a population of 10 million people at daytime. It has the highest MRRTDs in Iran [7]. The high population size, the natural diversity of the surrounding areas (windswept deserts, plains, and mountains) and the existence of industries have extensively increased the level of air pollution in this mega-city [8]. It is necessary to conduct a spatio-temporal analysis of the current epidemiological patterns of MRRTDs in Tehran to identify the potential links between MRRTDs and environmental risk factors. Spatio-temporal analyses of mortality data can provide new knowledge on spatial variation of MRRTDs and potential drivers of this variation. To support research in this field we aim to release the datasets of MRRTDs in Tehran, between 2008 and 2018.

### Data description

Geographical information system (GIS) has a great capacity to integrate diverse data from different sources including spatial, temporal and descriptive components into one framework. [9, 10]. Spatial component represents information about the physical location of health event and the shape of geometric objects. Temporal data refer to the time of occurrence and descriptive information relates to attribute data about the event (i.e. age, gender and cause of death) [11, 12]. GIS provides appropriate tools for collecting, geo-linking, analysing and visualising geographical patterns and spatio-temporal relationships in the distribution of RTDs or deaths with potential environmental factors [13, 14].

Data on 43,176 death events due to RTDs from September 2008 to September 2018 were obtained from the Behesht-e Zahra Organization, a local health department under the supervision of the Tehran Municipality [15]. This dataset includes date of death, age, gender, cause of death and urban district where death was occurred (dataset 1). Population data were obtained from the Statistical Centre of Iran and they contain the annual population size by district in Tehran from 2008 to 2018. We categorised the gender as male and female. Age of people was categorised into five groups including 0–14, 15–24, 25–44, 45–64 and > 65 years old (dataset 2). The digital boundaries of districts data (data file 3) and districts’ map sheets were obtained from the municipality of Tehran (data file 4) [16, 17]. Table 1 shows the details of each dataset and provides access links to these data.

**Table 1:** Overview of data sets

Label	Name of data file/data set	File types (file extension)	Data repository and identifier (DOI or accession number)
Dataset 1	Mortality data	MS Excel file (*.xlsx)	Harvard Dataverse https://doi.org/10.7910/DVN/NFKDWC
Dataset 2	Population data	MS Excel file (*.xlsx)	Harvard Dataverse https://doi.org/10.7910/DVN/NFKDWC
Data file 3	Tehran_border	Shape file (*.shp)	Harvard Dataverse https://doi.org/10.7910/DVN/NFKDWC
Data file 4	Tehran_districts	Shape file (*.shp)	Harvard Dataverse https://doi.org/10.7910/DVN/NFKDWC

The datasets provided in this study can be used by researchers in different disciplines such as health geography, urban planning, medicine and healthcare ecosystem research. Descriptive choropleth maps can be produced to highlight the MRRTDs distribution in Tehran during 2008–2018. Choropleth map is a common map used to represent data on predetermined geographic areas [18]. Researchers may use Empirical Bayesian procedures for smoothing mortality rates in the case of a choropleth map. Empirical Bayesian also help to identify local clusters of more/less affected areas [19]. High-mortality clusters (hot spots) show the regions having a significantly greater mortality rate and Low-mortality clusters reveal the regions with a significantly lower mortality rate (cold spots) in Tehran. Global Moran’s Index can be used to show the degree of spatial autocorrelation in the pattern of MRRTDs. In other words, it can test whether the mortalities distribute randomly or follow a spatial pattern [20].

In addition to spatio-temporal analyses, these data can be linked to the contextual factors such as climate status, air pollution and smoke to further investigated the impact of environmental risk factors on MRRTDs. The research outputs can inform health policymakers and urban planners to develop appropriate strategies to reduce the risk of MRRTDs across communities.

This study offers Tehran’s MRRTDs data for the period 2008–2018. Further, Covid-19 disease has become a pandemic disease in late 2019 and our data provide a new opportunity for researchers to quantify and assess the impact of Covid-19 disease on MRRTDs burden in Tehran.

## Limitations

Tehran is a mega city with an area of 730 km^2^ and comprises 22 districts. In this study, we aggregated the data at district level which is relatively a coarse geography and may mask variation at finer geography level such as mesh blocks.

## Data Availability

The data described in this Data note can be freely and openly accessed on Harvard Dataverse under https://doi.org/10.7910/DVN/NFKDWC [21]. Please see Table 1 and reference list for details and link to the data.

## Abbreviations

RTDs: Respiratory tract diseases
MRRTDs: Mortality rates due to respiratory tract diseases
GIS: Geographical information system
